# Co-recorded conditions in ischemic and hemorrhagic stroke: a 20-year national analysis of 598,186 natural deaths with machine learning

**DOI:** 10.3389/fneur.2026.1854246

**Published:** 2026-09-24

**Authors:** Junlin Ge, Hangang Jiang, Yu Fu, Hua Tian, Wencheng Yuan, Lingyun Sun

**Affiliations:** Department of Neurology, Yuechi County People’s Hospital, Guang’an City, Sichuan Province, China

**Keywords:** association rule mining, co-recorded condition, hemorrhagic stroke, ischemic stroke, machine learning, public health, stroke

## Abstract

The purpose of this study was to characterize the co-occurrence patterns of co-recorded conditions in ischemic and hemorrhagic stroke and identify co-recorded conditions using a large national dataset of fatal, subtype-confirmed stroke cases from Brazil. We analyzed 598,186 natural deaths recorded in the Brazilian Mortality Information System (SIM) between 2000 and 2019, comprising 288,724 (48.3%) hemorrhagic and 309,462 (51.7%) ischemic stroke. Hierarchical clustering was performed to identify co-recorded condition clusters, while association rule mining using the Apriori algorithm uncovered hidden patterns of disease co-occurrence. Logistic regression, random forest, and XGBoost models were developed to classify stroke subtype based on demographic characteristics and co-recorded condition profiles. Ischemic stroke patients had significantly higher co-recorded condition burden (median 2, IQR 1–3) compared to hemorrhagic stroke (median 1, IQR 1–2; *p* < 0.001). For hemorrhagic stroke, 12 association rules were identified; the strongest involved co-occurrence of infectious and respiratory diseases (e.g., {hypertension = Yes, infectious_disease = Yes} → {respiratory_disease = Yes}, lift = 2.57) and of endocrine/metabolic conditions with hypertension (lift = 1.67), whereas the rule {mental_disorders = Yes} → {hypertension = Yes} (lift = 1.27, confidence = 53.9%) was the weakest of the 12 rules. In contrast, ischemic stroke yielded 8 rules reflecting associations among infectious, respiratory, genitourinary, and endocrine/metabolic conditions. The logistic regression model achieved an AUC of 0.768 for predicting stroke subtype, with consistent performance across sensitivity analyses. Age-standardized analyses showed that ischemic stroke proportion increased from 48.8 to 51.5% over 20 years, with approximately half of the crude increase attributable to population aging (crude ischemic proportion reached 54.7%). Co-recorded condition burden increased substantially in both subtypes (≥3 co-recorded conditions in ischemic stroke: 24.8–33.3%). We observed that hemorrhagic and ischemic stroke have different co-occurrence patterns of co-recorded conditions: hemorrhagic stroke exhibited simpler co-occurrence patterns centered on hypertension together with infectious, respiratory, and endocrine/metabolic conditions, while ischemic stroke was characterized by clustered cardiovascular and metabolic multimorbidity. These findings warrant further investigation in prospective studies.

## 1 Introduction

Stroke remains a leading cause of mortality and long-term disability worldwide, with an estimated 12.2 million incident cases and 6.6 million deaths annually (1). The global burden of stroke has increased by 70% over the past three decades, driven largely by demographic transitions and the rising prevalence of cardiometabolic risk factors in low- and middle-income countries (LMICs) (2, 3). While ischemic stroke accounts for approximately 70% of all strokes globally, the proportion of hemorrhagic stroke remains substantially higher in LMICs compared to high-income countries, reflecting differences in hypertension control, healthcare infrastructure, and population demographics (4, 5). In Brazil, stroke is the second leading cause of death, with age-standardized mortality rates exceeding those of many high-income nations, yet comprehensive epidemiological data remain limited (6). From a public health perspective, understanding the determinants of stroke mortality is essential for developing effective population-level considerations and allocating limited healthcare resources, particularly in LMICs where the stroke burden is highest (4).

The relationship between co-recorded conditions and stroke subtype is increasingly recognized as critical for risk stratification, prevention, and prognostication. The INTERSTROKE study, a large international case–control study, identified 10 modifiable risk factors accounting for 90% of the population-attributable risk of stroke, with hypertension being the dominant risk factor for both subtypes (7). However, subsequent analyses revealed that risk factor profiles differ substantially between ischemic and hemorrhagic stroke, with metabolic and cardiovascular conditions more strongly associated with ischemic stroke, while hypertension and alcohol use show stronger associations with hemorrhagic stroke (8, 9). Recent studies have demonstrated distinct co-recorded condition patterns associated with each subtype, with cardiovascular and metabolic conditions clustering in ischemic stroke and hypertension remaining the predominant factor in hemorrhagic stroke (10, 11). These findings have direct implications for public health policy, as they suggest that subtype-specific considerations may be more effective than a one-size-fits-all approach, enabling more efficient allocation of resources in resource-limited settings (4, 12).

Despite these advances, significant knowledge gaps persist that have direct relevance to public health practice and policy. First, most research has focused on high-income populations, with limited data from LMICs where the epidemiological transition is most dynamic and the burden of stroke is highest (4, 12). Addressing this gap is critical for achieving the United Nations Sustainable Development Goal 3 (SDG 3) of ensuring healthy lives and promoting wellbeing for all at all ages, particularly in middle-income countries undergoing rapid epidemiological transition (4). Second, existing studies have typically examined co-recorded conditions in isolation rather than exploring the complex, multidimensional patterns of disease co-occurrence that characterize real-world clinical populations (13, 14). Understanding these patterns at the population level can inform integrated care models, guide resource allocation, and support the development of community-based interventions that address multimorbidity rather than treating conditions in isolation (10, 13). Third, the role of mental disorders in stroke pathogenesis remains particularly understudied, despite growing evidence linking psychological stress, depression, and anxiety to cardiovascular events through neuroendocrine and inflammatory pathways (15, 16). From a public mental health perspective, this gap is concerning given the high and increasing prevalence of mental disorders worldwide and the well-documented disparities in mental health care access across LMICs (17, 18). A recent large cohort study found that psychological distress was associated with a 27% increased risk of stroke, with stronger associations observed for hemorrhagic stroke (19). However, few studies have systematically examined the co-occurrence of mental disorders with traditional cardiovascular risk factors across stroke subtypes. Identifying such associations could inform integrated mental health and cardiovascular disease prevention considerations at the community level, addressing both physical and mental health as part of comprehensive primary care (4, 15). We acknowledge, however, that death certificate data have inherent limitations in capturing chronic psychological conditions compared to prospective cohort studies with standardized diagnostic instruments, and may preferentially identify severe or institutionalized cases.

To address these gaps and provide evidence for public health policy and practice, we leveraged a large national dataset of 598,186 natural deaths in Brazil between 2000 and 2019 to characterize the co-occurrence patterns of co-recorded conditions in ischemic and hemorrhagic stroke. The Brazilian SIM database was selected because it offers a large, nationally comprehensive, publicly available dataset spanning two decades (2000–2019), providing a unique opportunity to characterize stroke co-recorded condition patterns at population scale in a middle-income country undergoing rapid epidemiological transition. Using a combination of hierarchical clustering, association rule mining, and machine learning approaches, we aimed to: (1) identify distinct co-recorded condition patterns associated with each stroke subtype; (2) investigate co-occurrence patterns, including mental disorders; and (3) examine temporal trends in co-recorded condition burden over two decades. This study directly supports SDG 3 by providing evidence for targeted considerations in a middle-income country undergoing epidemiological transition, with findings that may offer insights into fatal stroke patterns in other middle-income countries with comparable demographic and health transitions, acknowledging the diversity across LMIC settings (4, 6, 20). Our findings provide a comprehensive characterization of stroke co-recorded condition patterns in Latin America, generating hypotheses for future research in stroke epidemiology (5, 12).

Several prior studies have applied machine learning to stroke prediction. Heo et al. (21) developed ML models for acute stroke outcome prediction, while Chen et al. (22) used ML approaches for mortality prediction in critically ill stroke patients. However, these studies focused on clinical outcomes rather than co-occurrence patterns of co-recorded conditions. In Brazil, de Souza et al. (23) recently analyzed stroke death profiles from the same SIM database using decision tree models, but did not examine co-recorded condition co-occurrence patterns or mental disorders as specific associated conditions. Multimorbidity research in stroke has primarily examined individual risk factors in isolation (10, 11), with limited application of unsupervised methods such as association rule mining to characterize disease co-occurrence patterns (14). Our study extends this literature by: (i) applying association rule mining to a large national dataset of fatal stroke cases from an LMIC setting; (ii) systematically comparing co-occurrence patterns of co-recorded conditions between ischemic and hemorrhagic stroke; and (iii) identifying mental disorders co-occurrence as a hypothesis-generating signal for hemorrhagic stroke.

## 2 Methods

### 2.1 Data source and study population

We extracted individual-level mortality records from the Brazilian Mortality Information System (SIM) between 2000 and 2019. Of approximately 2.46 million death records mentioning cerebrovascular disease (ICD-10 I60–I69), after sequentially excluding unspecified stroke subtype (I64), multiple stroke subtypes, age <19 years, missing data, and deaths from external causes (*n* = 33,886; total exclusions: 1,861,556), the final study population comprised 598,186 eligible natural deaths, including 288,724 (48.3%) hemorrhagic (I60–I62) and 309,462 (51.7%) ischemic stroke (I63) (23, 24). The study population selection process is illustrated in Figure 1. The dataset analyzed in this study is publicly available (25).

**Figure 1:**
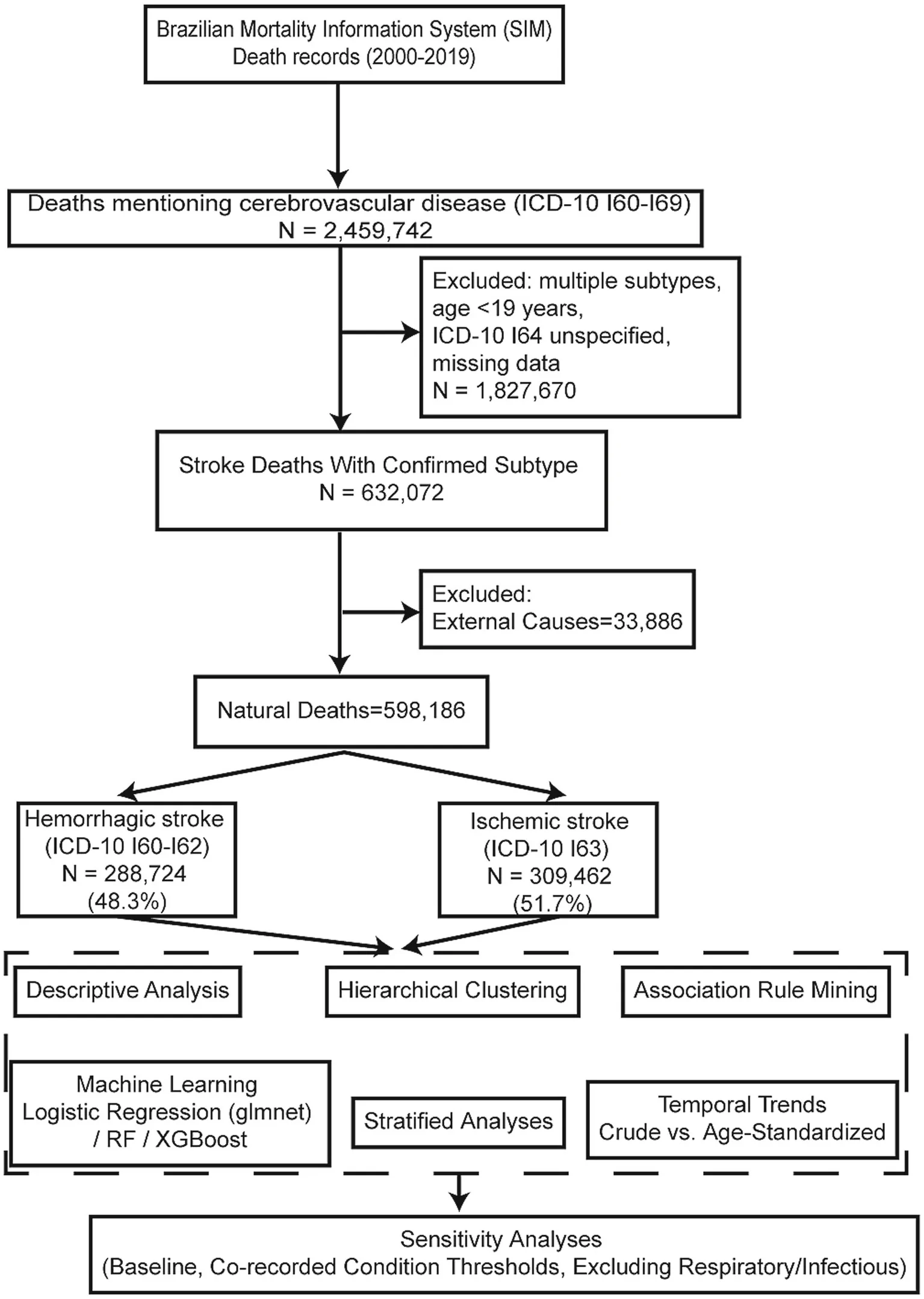
Study design and analysis workflow. End-to-end methodology from data extraction to final results.

### 2.2 Variables and definitions

The primary outcome was stroke subtype: hemorrhagic (ICD-10 I60-I62) or ischemic (I63). Unspecified stroke (I64), which accounted for approximately 62% of all cerebrovascular death mentions in the original SIM database (25), was excluded from main analyses. This exclusion may introduce selection bias toward cases with confirmed subtype, which are more likely to occur in regions or time periods with greater CT/MRI availability.

Demographic variables included sex, race/ethnicity, age group (19–39, 40–59, 60–79, ≥80 years), education, marital status, geographic region (North, Northeast, Southeast, South, Central-West), and place of death. The two youngest age groups (19–39 and 40–59 years) were combined into a single <60 years category in the age-stratified analyses.

Fourteen co-recorded conditions were identified from death certificates, including hypertension, respiratory disease, endocrine/metabolic disorders, cardiovascular conditions (e.g., heart failure, arrhythmias, ischemic heart disease), infectious disease, and mental disorders, among others. Each co-recorded condition was coded as present or absent based on ICD-10 codes (detailed in Supplementary Table S1). Mental disorders encompassed the full ICD-10 F00–F99 range, including dementia (F00–F03), substance use disorders (F10–F19), schizophrenia (F20–F29), and mood disorders (F30–F39), among others. However, the available data record mental disorders only as a single binary variable without reliable disaggregation into individual ICD-10 subchapters, precluding separate analysis of substance-induced versus non-substance psychiatric disorders. It is important to note that in death certificate data, pre-existing conditions cannot be reliably distinguished from acute complications of stroke. Therefore, all recorded conditions are treated as contemporaneous co-recorded diagnoses.

### 2.3 Statistical analysis

Analyses were performed using R version 4.4.0, with two-sided *p*-values <0.05 considered statistically significant. Demographic and clinical characteristics were compared between stroke subtypes using chi-square tests (categorical variables) and Wilcoxon rank-sum tests (continuous variables). Co-recorded condition burden was quantified as the total number of co-recorded conditions per individual. Results are presented as means (SD) or medians (IQR) for continuous variables, and frequencies (percentages) for categorical variables.

### 2.4 Co-recorded condition clustering

Hierarchical clustering with Ward’s linkage and Euclidean distance was used to group co-recorded conditions by their prevalence patterns across stroke subtypes (26). The number of clusters (*k* = 4) was selected based on dendrogram structure (average silhouette width = 0.533) (27). Pairwise correlations between binary co-recorded conditions were calculated using the phi coefficient (equivalent to Pearson correlation for binary variables) and visualized as a correlation matrix (Figure 2C). Clusters were visualized using dendrograms and heatmaps. The clustering was based solely on the prevalence profiles of the 14 co-recorded conditions across the two stroke subtypes; the phi correlation matrices (Figure 2C) were generated separately to visualize pairwise co-occurrence structure and were not inputs to the clustering (13). Accordingly, the identified clusters describe similarity in conditions’ prevalence patterns across the two stroke subtypes and do not represent patient-level multimorbidity clusters.

**Figure 2:**
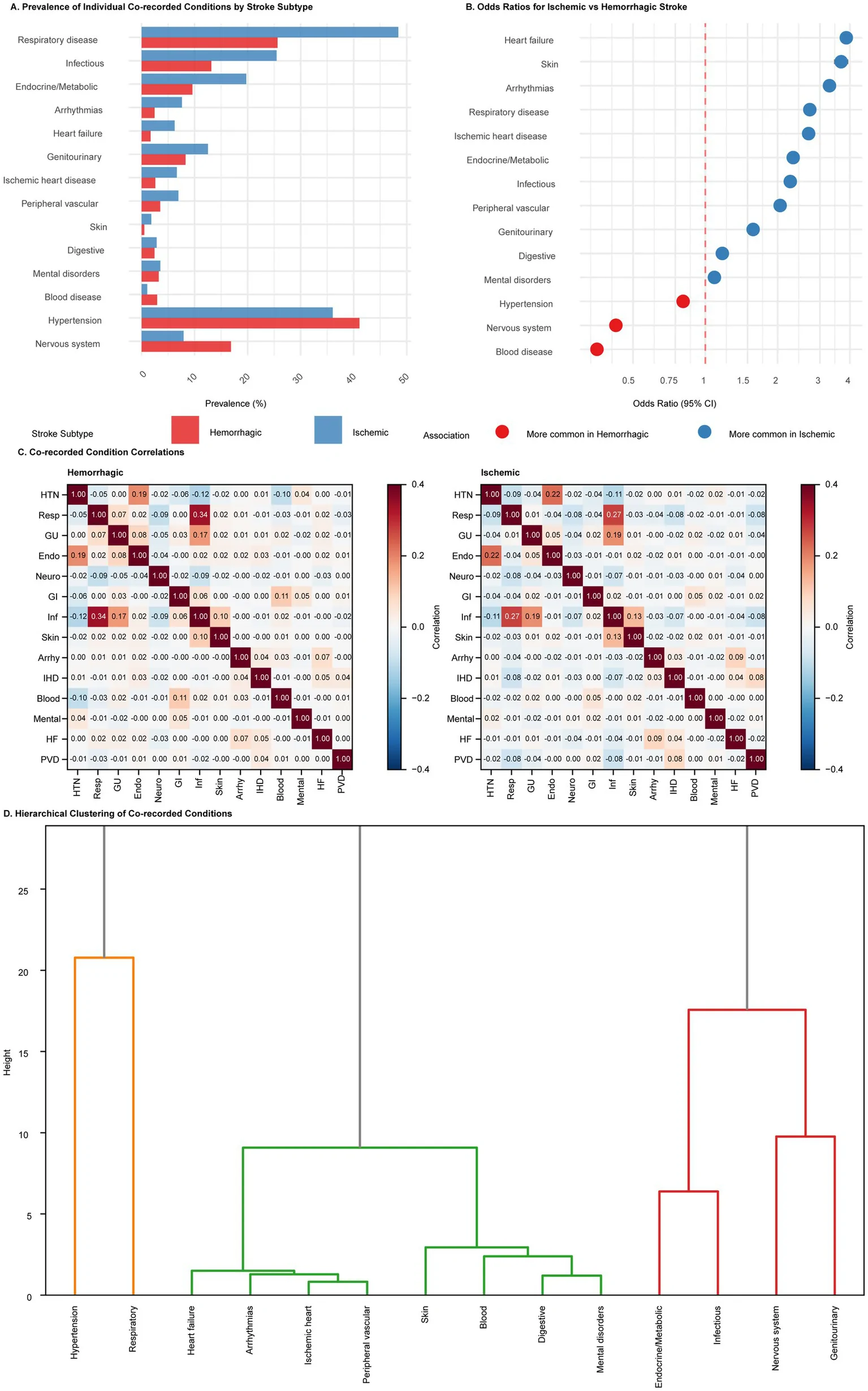
Co-recorded condition patterns: **(A)** Prevalence of individual co-recorded conditions. **(B)** Forest plot of odds ratios (ischemic vs. hemorrhagic). Absolute prevalence values for each co-recorded condition are presented in Table 3. **(C)** Correlation matrices (ischemic and hemorrhagic stroke). **(D)** Hierarchical clustering dendrogram showing four clusters of co-recorded conditions grouped by similar prevalence patterns across stroke subtypes.

### 2.5 Association rule mining

We applied the Apriori algorithm to uncover co-recorded condition co-occurrence patterns separately for each stroke subtype (excluding external causes) (28). The minimum support threshold was set to 0.5% for hemorrhagic stroke and 2% for ischemic stroke. The lower threshold for hemorrhagic stroke was necessary because the joint support of multi-condition co-occurrence patterns was substantially lower among hemorrhagic decedents, who had fewer co-recorded conditions overall (median 1 vs. 2); a uniform 2% threshold would have failed to capture most multi-item associations, which typically occurred in fewer than 2% of hemorrhagic decedents. Rules were filtered using a minimum confidence of 50% and a lift threshold of >1, indicating positive association (29). Rule length was restricted to 2–4 items to balance interpretability and informativeness; longer rules become difficult to interpret clinically and are more prone to overfitting. The Apriori algorithm was chosen over alternatives such as FP-Growth because of its simplicity, interpretability, and efficiency with sparse binary transaction data of this type, where the total number of items (co-recorded conditions) is relatively small. Rules were ranked by lift and visualized using grouped plots and network graphs (30). To ensure that the reported rules reflect positive co-occurrence, only rules in which all co-recorded conditions were present (i.e., items coded ‘=Yes’) were retained for Table 1; Figure 3; rules involving the absence of a condition (‘=No’ items) were excluded. The lower support threshold for hemorrhagic stroke (0.5% vs. 2%) increases sensitivity to detect patterns but may also introduce spurious associations. Consequently, the resulting rule counts are not directly comparable across subtypes.

**Table 1:** Top association rules by stroke subtype.

Subtype	Rule	Support (%)	Confidence (%)	Lift
Hemorrhagic	{hypertension = Yes + infectious_disease = Yes → respiratory_disease = Yes}	2.34	65.1	2.57
Hemorrhagic	{infectious_disease = Yes → respiratory_disease = Yes}	8.28	63.4	2.5
Hemorrhagic	{endocrine_metabolic_disease = Yes + hypertension = Yes + infectious_disease = Yes → respiratory_disease = Yes}	0.52	63.1	2.49
Hemorrhagic	{endocrine_metabolic_disease = Yes + infectious_disease = Yes → respiratory_disease = Yes}	0.92	61.4	2.42
Hemorrhagic	{genitourinary_disease = Yes + infectious_disease = Yes → respiratory_disease = Yes}	1.39	51.8	2.04
Hemorrhagic	{endocrine_metabolic_disease = Yes → hypertension = Yes}	6.89	71.0	1.67
Hemorrhagic	{endocrine_metabolic_disease = + nervous_system_disease = Yes → hypertension = Yes}	0.85	69.5	1.64
Hemorrhagic	{endocrine_metabolic_disease = Yes + respiratory_disease = Yes → hypertension = Yes}	1.7	62.3	1.47
Hemorrhagic	{endocrine_metabolic_disease = Yes + genitourinary_disease = Yes → hypertension = Yes}	0.89	60.0	1.41
Hemorrhagic	{endocrine_metabolic_disease = Yes + infectious_disease = Yes + respiratory_disease = Yes → hypertension = Yes}	0.52	56.3	1.33
Hemorrhagic	{endocrine_metabolic_disease = Yes + infectious_disease = Yes → hypertension = Yes}	0.82	54.7	1.29
Hemorrhagic	{mental_disorders = Yes → hypertension = Yes}	1.75	53.9	1.27
Ischemic	{genitourinary_disease = Yes + respiratory_disease = Yes → infectious_disease = Yes}	3.15	51.6	2.05
Ischemic	{endocrine_metabolic_disease = Yes → hypertension = Yes}	11.49	58.1	1.6
Ischemic	{endocrine_metabolic_disease = Yes + respiratory_disease = Yes → hypertension = Yes}	4.77	54.2	1.49
Ischemic	{infectious_disease = Yes → respiratory_disease = Yes}	18.06	71.6	1.49
Ischemic	{hypertension = Yes + infectious_disease = Yes → respiratory_disease = Yes}	4.92	71.4	1.48
Ischemic	{endocrine_metabolic_disease = Yes + infectious_disease = Yes → hypertension = Yes}	2.48	51	1.4
Ischemic	{endocrine_metabolic_disease = Yes + infectious_disease = Yes → respiratory_disease = Yes}	3.27	67.1	1.39
Ischemic	{genitourinary_disease = Yes + infectious_disease = Yes → respiratory_disease = Yes}	3.15	54.5	1.13

Rules ranked by lift within each subtype. Only rules in which all co-recorded conditions were present (‘=Yes’ items) are shown. Support = percentage of patients satisfying the rule. Confidence = probability of consequent given antecedent. Lift >1 indicates positive association.

**Figure 3:**
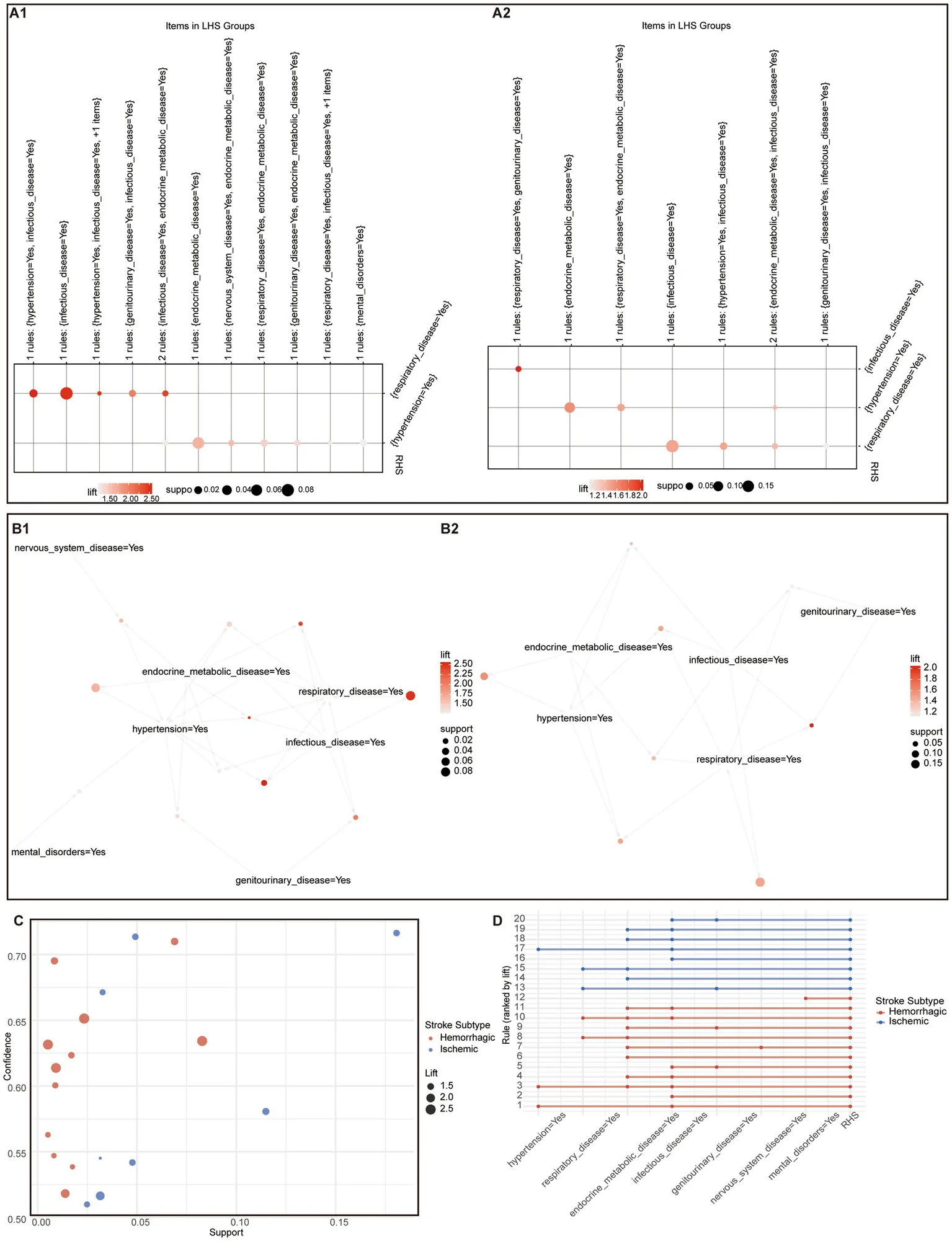
Association rule mining results: **(A1)** Grouped rule plot for hemorrhagic stroke (12 rules). **(A2)** Grouped rule plot for ischemic stroke (8 rules). **(B1)** Network graph for hemorrhagic stroke. **(B2)** Network graph for ischemic stroke. **(C)** Scatter plot of all 20 rules (support × confidence), color-coded by stroke subtype (red: hemorrhagic; blue: ischemic), with point size proportional to lift. **(D)** Parallel coordinates plot of all rules color-coded by stroke subtype. In panels A1 and A2, rows annotated “2 rules” contain two rules sharing the same antecedent. Only rules in which all co-recorded conditions were present are shown; the rules correspond to those listed in Table 1.

### 2.6 Machine learning models

To quantify the discriminative capacity of co-recorded condition profiles beyond descriptive analyses, three machine learning models were developed to classify stroke subtype using demographics and 14 co-recorded conditions. Data were split into training (70%) and testing (30%) sets, with stratification by stroke subtype to preserve class proportions in both subsets. Ischemic stroke was set as the reference outcome; thus, positive coefficients indicate a higher likelihood of ischemic stroke. Threshold-dependent metrics (accuracy, sensitivity, specificity, PPV, NPV) were computed with hemorrhagic stroke as the positive class and evaluated at the default 0.5 decision threshold. 95% confidence intervals were estimated from 1,000 bootstrap iterations of the test set.

Logistic regression with L2 regularization was fitted using glmnet (31) with 5-fold cross-validation. Random forest (100 trees, subsampled to 30,000 observations drawn from the 418,730 training records, ~7%, for computational efficiency) was implemented using randomForest (32), with feature importance assessed by mean decrease in Gini. XGBoost was implemented using xgboost (33) with the following hyperparameters: max_depth = 6, eta = 0.3, nrounds = 100, subsample = 0.8, colsample_bytree = 0.8, with 5-fold cross-validation for early stopping. Categorical variables (sex, age group, region, education) were encoded as dummy variables. No explicit class imbalance handling was required because the outcome classes were approximately balanced (48.3% hemorrhagic vs. 51.7% ischemic). Performance was evaluated using accuracy, sensitivity, specificity, and AUC. Hyperparameters for XGBoost were selected empirically; no exhaustive grid search was performed.

### 2.7 Sensitivity analyses

Five sensitivity analyses tested robustness: (1) baseline (natural deaths only); (2) restricting to deaths with ≥1 co-recorded condition; (3) restricting to deaths with ≥2 co-recorded conditions; (4) case restriction excluding deaths with respiratory disease recorded on the death certificate (*N* = 375,700); and (5) case restriction excluding deaths with infectious disease recorded on the death certificate (*N* = 482,451). In the case-restriction analyses, because the excluded decedents all had the respective condition recorded, the corresponding indicator variable became constant (coded ‘No’) among the remaining deaths and was removed from the predictor set; the models therefore used the demographic variables together with the remaining 13 co-recorded conditions.

### 2.8 Stratified analyses

Age-stratified (young <60, middle-aged 60–79, elderly ≥80 years) and sex-stratified analyses were conducted, repeating logistic regression and association rule mining for each stratum. For the age-stratified analyses, the 19–39 and 40–59 years descriptive groups were combined into a single <60 years category to ensure adequate sample sizes within strata; the 60–79 and ≥80 years groups were retained unchanged. The <60 years group therefore corresponds to the combined two youngest descriptive age groups, which are presented separately in the descriptive analyses.

### 2.9 Temporal trend analysis

Four 5-year periods (2000–2004 to 2015–2019) were analyzed. Trends in stroke subtype proportions, co-recorded condition prevalence, and co-recorded condition burden were assessed by calculating percent change from the first (2000–2004) to the last (2015–2019) period, comparing crude and age-standardized estimates (34).

### 2.10 Missing data and ethics

Missing data were minimal (<1%); complete case analysis was performed. The study used de-identified public data and was exempt from institutional review board approval.

## 3 Results

### 3.1 Study population and co-recorded condition burden

A total of 598,186 natural deaths were included, comprising 288,724 (48.3%) hemorrhagic and 309,462 (51.7%) ischemic stroke (Table 2). Ischemic stroke patients were significantly older (85.6% aged ≥60 years vs. 57.6%, *p* < 0.001), while hemorrhagic stroke predominated in younger individuals (42.4% under 60 years vs. 14.4%). Sex distribution was similar between subtypes (51.0% female in ischemic vs. 50.8% in hemorrhagic, *p* < 0.001). Regional variations showed higher hemorrhagic stroke proportions in the North (56.1% 16,539/29,497) and Northeast (50.7% 56,410/111,177), while ischemic stroke predominated in the South (59.8% 69,946/116,854; *p* < 0.001).

**Table 2:** Demographic and clinical characteristics by stroke subtype.

Characteristic	Total (*N* = 598,186)	Hemorrhagic (*n* = 288,724)	Ischemic (*n* = 309,462)	*p*-value
Age group *n* (%)				<0.001
19–39 years	29,999 (5.0)	24,563 (8.5)	5,436 (1.8)	
40–59 years	137,054 (22.9)	97,884 (33.9)	39,170 (12.7)	
60–79 years	265,118 (44.3)	116,796 (40.5)	148,322 (47.9)	
≥80 years	166,015 (27.8)	49,481 (17.1)	116,534 (37.7)	
Sex *n* (%)				<0.001
Female	304,498 (50.9)	146,653 (50.8)	157,845 (51.0)	
Male	293,688 (49.1)	142,071 (49.2)	151,617 (49.0)	
Region *n* (%)				<0.001
North	29,497 (4.9)	16,539 (5.7)	12,958 (4.2)	
Northeast	111,177 (18.6)	56,410 (19.5)	54,767 (17.7)	
Southeast	298,182 (49.8)	147,704 (51.2)	150,478 (48.6)	
South	116,854 (19.5)	46,908 (16.2)	69,946 (22.6)	
Central-West	42,476 (7.1)	21,163 (7.3)	21,313 (6.9)	
Education *n* (%)				<0.001
None	120,798 (20.2)	47,039 (16.3)	73,759 (23.8)	
1–3 years	196,478 (32.8)	87,506 (30.3)	108,972 (35.2)	
4–7 years	154,307 (25.8)	79,599 (27.6)	74,708 (24.1)	
8–11 years	87,618 (14.6)	51,241 (17.7)	36,377 (11.8)	
≥12 years	38,985 (6.5)	23,339 (8.1)	15,646 (5.1)	
Co-recorded condition count median (IQR)	2 (1–3)	1 (1–2)	2 (1–3)	<0.001

Data are presented as *n* (%) unless otherwise indicated. *p*-values were calculated using chi-square tests for categorical variables (age group, sex, region, education) and Wilcoxon rank-sum test for co-recorded condition count. IQR, interquartile range.

Ischemic stroke patients had significantly higher co-recorded condition burden (median 2, IQR 1–3) compared to hemorrhagic stroke (median 1, IQR 1–2; *p* < 0.001; Figure 4). The percentage distribution (Figure 4B) shows that 29.6% of ischemic stroke patients had ≥3 co-recorded conditions, nearly double the proportion in hemorrhagic stroke (14.9%). Conversely, hemorrhagic stroke patients were more likely to have no co-recorded conditions (22.4% vs. 10.8%). Notably, hypertension, nervous system disease, and blood disease were the only co-recorded conditions with higher death certificate documentation in hemorrhagic stroke (hypertension: 42.5% vs. 36.4%; nervous system disease: 16.9% vs. 7.9%; blood disease: 2.9% vs. 1.0%).

**Figure 4:**
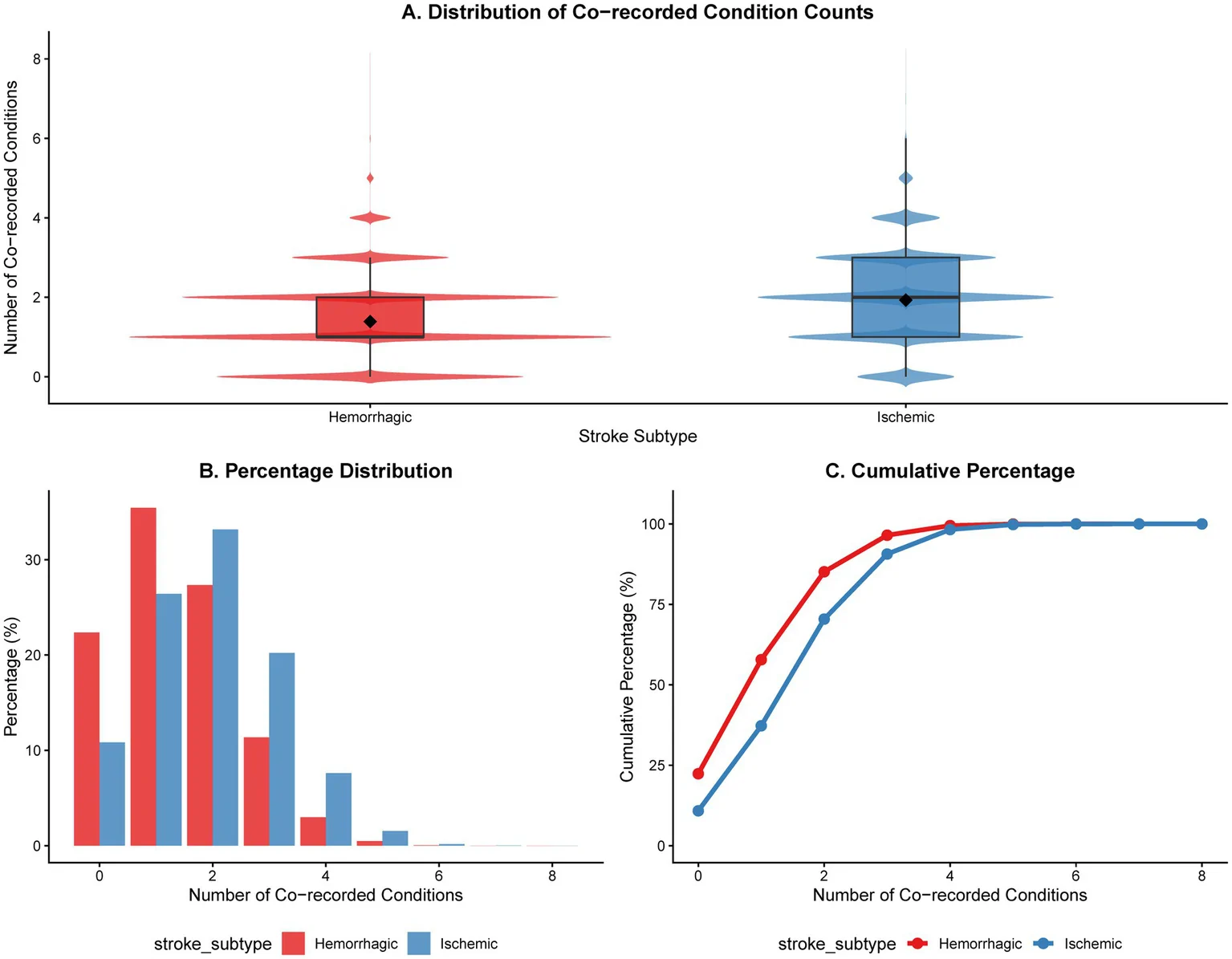
Distribution of co-recorded condition counts by stroke subtype. **(A)** Boxplot and violin plot of co-recorded condition counts in hemorrhagic and ischemic stroke. **(B)** Percentage distribution of co-recorded condition counts (0, 1, 2, ≥3). **(C)** Cumulative percentage of co-recorded condition counts by stroke subtype.

This pattern persisted across all age strata. Among patients aged <60 years, ischemic stroke decedents had a higher mean co-recorded condition count (1.79 vs. 1.24) and a greater proportion with ≥3 co-recorded conditions (25.6% vs. 11.7%) compared to hemorrhagic stroke. Similar patterns were observed in the 60–79 and ≥80 age groups (Supplementary Table S2).

### 3.2 Co-recorded condition patterns and clustering

All 14 co-recorded conditions differed significantly between stroke subtypes (Table 3, all *p* < 0.001). Ischemic stroke patients had markedly higher prevalence of respiratory disease (48.2% vs. 25.4%), infectious disease (25.2% vs. 13.1%), endocrine/metabolic disorders (19.8% vs. 9.7%), and cardiovascular conditions such as heart failure (6.3% vs. 1.7%) and arrhythmias (7.6% vs. 2.4%; Figure 2). The forest plot (Figure 2B) confirms strong associations with ischemic stroke for cardiovascular and metabolic conditions (heart failure: OR = 3.86; arrhythmias: OR = 3.40).

**Table 3:** Prevalence of individual co-recorded conditions by stroke subtype.

Co-recorded condition	Hem (%)	Isch (%)	OR (95% CI)	Age-adj OR (95% CI)
Hypertension	42.5	36.4	0.77 (0.77–0.78)	0.73 (0.73–0.74)
Respiratory disease	25.4	48.2	2.73 (2.72–2.75)	2.35 (2.34–2.36)
Genitourinary	8.2	12.4	1.58 (1.57–1.60)	1.52 (1.50–1.53)
Endocrine/metabolic	9.7	19.8	2.29 (2.28–2.31)	2.13 (2.12–2.15)
Nervous system disease	16.9	7.9	0.42 (0.42–0.43)	0.49 (0.48–0.49)
Digestive	2.4	2.7	1.15 (1.13–1.17)	1.27 (1.25–1.29)
Infectious	13.1	25.2	2.24 (2.23–2.26)	2.07 (2.06–2.09)
Skin	0.5	1.8	3.66 (3.58–3.75)	3.06 (2.99–3.14)
Arrhythmias	2.4	7.6	3.40 (3.36–3.44)	2.74 (2.71–2.77)
Ischemic heart disease	2.5	6.5	2.65 (2.61–2.68)	2.42 (2.39–2.45)
Blood disease	2.9	1	0.36 (0.35–0.36)	0.43 (0.42–0.44)
Mental disorders	3.3	3.5	1.08 (1.07–1.10)	1.37 (1.35–1.39)
Heart failure	1.7	6.3	3.86 (3.82–3.91)	3.12 (3.08–3.16)
Peripheral vascular disease	3.3	6.8	2.12 (2.09–2.14)	1.97 (1.95–2.00)

Hem (%), prevalence of each co-recorded condition among hemorrhagic stroke decedents; Isch (%), prevalence among ischemic stroke decedents. OR, odds ratio (ischemic vs. hemorrhagic); CI, confidence interval. All *p*-values <0.001.

Correlation matrices (Figure 2C) showed moderate positive correlations between respiratory and infectious diseases in ischemic stroke (*r* = 0.27), while pairwise correlations among cardiovascular conditions were minimal (hypertension–heart failure *r* = −0.01; hypertension–arrhythmias *r* = 0.00). Hierarchical clustering, based on prevalence profiles of the 14 conditions across subtypes, identified four distinct clusters (average silhouette width = 0.533), grouping conditions with similar prevalence patterns across subtypes (e.g., respiratory and infectious diseases; cardiovascular and metabolic conditions; neuropsychiatric conditions; Figure 2D); these clusters reflect similarity in prevalence patterns and do not represent patient-level multimorbidity clusters.

### 3.3 Association rule mining

Association rule mining identified 12 rules for hemorrhagic stroke and 8 for ischemic stroke (Table 1; Figure 3). Because different minimum support thresholds were applied (0.5% for hemorrhagic stroke vs. 2% for ischemic stroke), the number of rules and the complexity of the resulting networks are not directly comparable between subtypes. For hemorrhagic stroke, the strongest rules involved co-occurrence of infectious and respiratory diseases (e.g., {hypertension = Yes, infectious_disease = Yes} → {respiratory_disease = Yes}, lift = 2.57; {infectious_disease = Yes} → {respiratory_disease = Yes}, lift = 2.50) and of endocrine/metabolic conditions with hypertension (e.g., {endocrine_metabolic_disease = Yes} → {hypertension = Yes}, lift = 1.67); the rule {mental_disorders = Yes} → {hypertension = Yes} (support = 1.75%, lift = 1.27, confidence = 53.9%) was the weakest of the 12 hemorrhagic rules (Table 1). Ischemic stroke rules showed moderate lift (range: 1.13–2.05), reflecting associations among infectious, respiratory, genitourinary, and endocrine/metabolic conditions (Table 1). Network visualization (Figures 3C,D) illustrated these co-occurrence patterns. Only rules in which all co-recorded conditions were present are shown; rules involving the absence of a condition were excluded.

### 3.4 Machine learning classification

Logistic regression and XGBoost achieved comparable performance (AUC = 0.768 and 0.773, respectively; accuracy 70.6–70.8%), outperforming Random Forest (AUC = 0.755; Table 4; Figure 5). The top predictors (Figure 6) were hypertension, age group, respiratory disease, endocrine/metabolic disease, heart failure, and arrhythmias. The logistic regression model demonstrated good calibration (Figure 6B), with predicted probabilities lying close to the ideal diagonal line. Given the minimal AUC difference and the importance of interpretability, logistic regression was used for subsequent analyses.

**Table 4:** Machine learning model performance.

Model	Accuracy	Sensitivity	Specificity	PPV	NPV	AUC
Logistic regression	0.706 (0.703–0.708)	0.672 (0.669–0.675)	0.737 (0.734–0.740)	0.704	0.707	0.768 (0.766–0.771)
Random forest	0.698 (0.695–0.699)	0.660 (0.657–0.663)	0.732 (0.729–0.735)	0.697	0.698	0.755 (0.753–0.758)
XGBoost	0.708 (0.706–0.710)	0.656 (0.653–0.659)	0.757 (0.754–0.759)	0.715	0.702	0.773 (0.771–0.775)

PPV, positive predictive value; NPV, negative predictive value; AUC, area under the ROC curve. Logistic regression was fitted as an L2-penalized model using glmnet. Hemorrhagic stroke was the positive class; metrics were evaluated at the 0.5 decision threshold. All three models were trained on 70% (*n* = 418,730) and tested on 30% (*n* = 179,456). Random forest was trained on a 30,000-observation subsample of the training set and tested on the same 30% (*n* = 179,456) held-out set.

**Figure 5:**
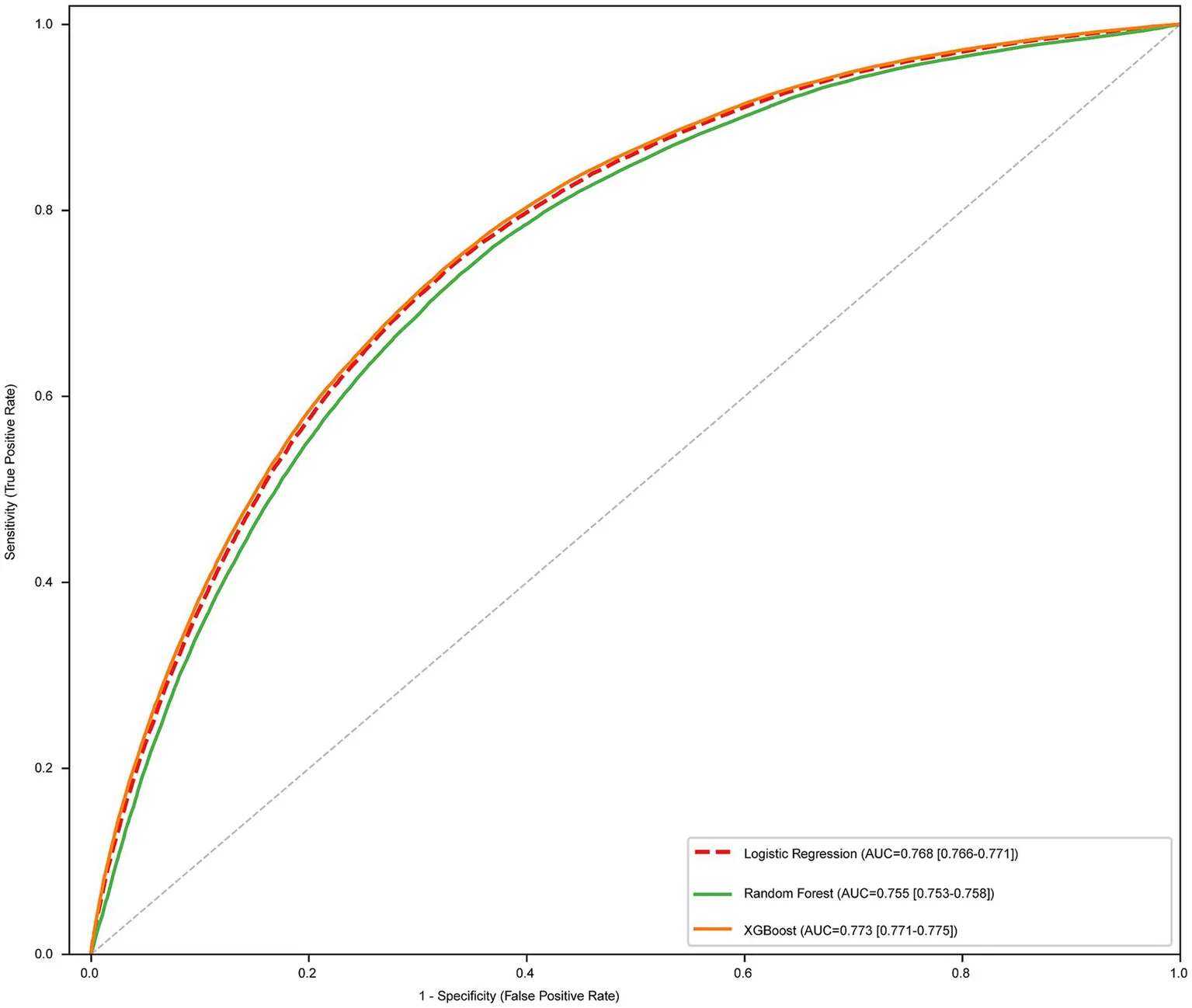
ROC curves for stroke subtype prediction: ROC curves for logistic regression (L2-penalized, fitted via glmnet), random forest, and XGBoost. Diagonal dashed line represents chance performance (AUC = 0.5).

**Figure 6:**
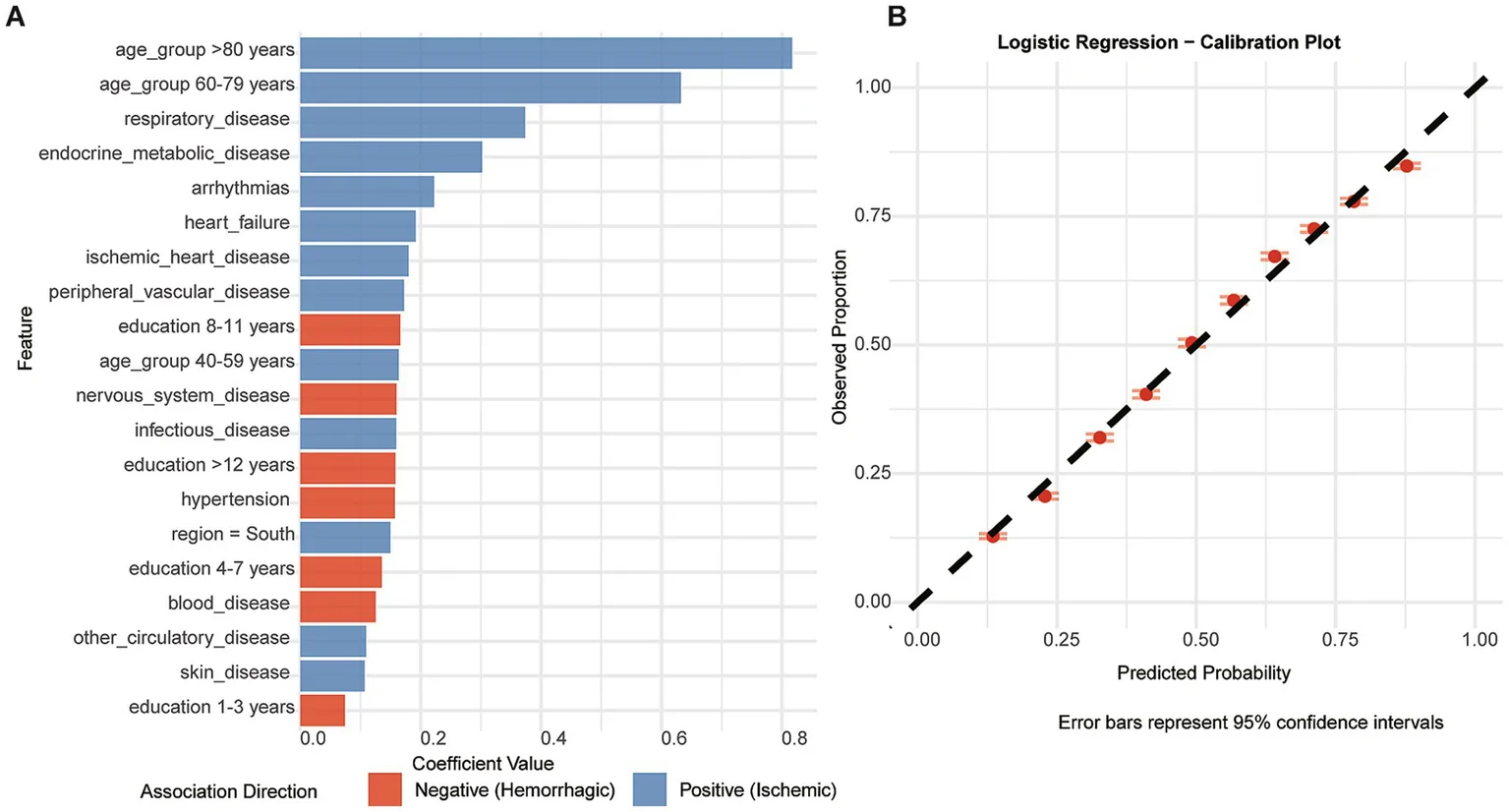
Feature importance and model calibration **(A)** Top 20 predictors from logistic regression (coefficient values); positive coefficients indicate association with ischemic stroke and negative coefficients with hemorrhagic stroke. **(B)** Calibration plot showing predicted vs. observed probabilities. Dashed line indicates perfect calibration.

### 3.5 Sensitivity and stratified analyses

The logistic regression model demonstrated stable performance across five sensitivity analyses, with AUC values ranging from 0.740 to 0.772 (Table 5; Supplementary Figure S1). Because sensitivity and specificity were evaluated at a fixed 0.5 threshold, their variation across sensitivity analyses reflects differences in case mix and predicted probabilities after each restriction, while AUC remained stable (0.740–0.772), indicating largely unchanged discriminative performance. The {mental_disorders = Yes} → {hypertension = Yes} rule identified in the main analysis was examined across all sensitivity analyses (Supplementary Table S5). In the ≥1 co-recorded condition subset, the lift of this rule was exactly 0.98 (below 1), indicating no positive association in that subset; the rule was therefore not retained there under the lift >1 criterion.

**Table 5:** Sensitivity analyses of model performance.

Dataset	N	Accuracy	Sensitivity	Specificity	AUC
Natural deaths only (baseline)	598,186	0.706 (0.703–0.708)	0.672 (0.669–0.676)	0.737 (0.734–0.740)	0.768 (0.766–0.771)
≥1 co-recorded condition	500,074	0.709 (0.707–0.711)	0.613 (0.609–0.617)	0.787 (0.784–0.790)	0.764 (0.762–0.767)
≥2 co-recorded conditions	315,991	0.710 (0.707–0.712)	0.466 (0.461–0.471)	0.862 (0.860–0.865)	0.740 (0.737–0.743)
Excluding respiratory disease	375,700	0.705 (0.702–0.708)	0.795 (0.792–0.799)	0.583 (0.579–0.588)	0.763 (0.761–0.766)
Excluding infectious disease	482,451	0.706 (0.703–0.708)	0.723 (0.719–0.725)	0.687 (0.684–0.691)	0.772 (0.770–0.775)

N indicates the full cohort size of each sensitivity dataset before the 70/30 training-test split; all metrics were evaluated on the corresponding held-out test set. CI, confidence interval. All sensitivity analyses used the same logistic regression model with demographics and co-recorded conditions as predictors (all 14 conditions in the baseline, ≥1-condition, and ≥2-condition analyses; the remaining 13 conditions in the case-restriction analyses, where the excluded condition became constant and was removed). Hemorrhagic stroke was the positive class; metrics evaluated at the 0.5 threshold. 95% CI: 1,000 bootstrap iterations of each respective test set.

Age-stratified analysis showed that hemorrhagic stroke predominated in young patients (73.3%) but declined to 44.1% in middle-aged patients (Supplementary Figure S2). The model showed high sensitivity (94.9%) but low specificity (22.6%) in young patients (AUC = 0.717), while middle-aged patients demonstrated more balanced performance (AUC = 0.710). Sex-stratified analysis showed higher model performance in females (AUC = 0.748) compared to males (AUC = 0.727; Supplementary Figure S3). Regional analysis (Supplementary Figure S4) confirmed that hemorrhagic stroke proportion was highest in the North (56.1%) and lowest in the South (40.1%), with the {mental_disorders = Yes} → {hypertension = Yes} rule also identified across regions (lift range: 1.21–1.32). This suggests that the observed co-recorded condition patterns are not driven solely by regional differences in diagnostic capacity. Demographic profiles and stratified model performance by age and sex are detailed in Supplementary Tables S2–S4, and the robustness of the key association rule is shown in Supplementary Table S5.

### 3.6 Temporal trends (2000–2019)

The crude proportion of ischemic stroke increased from 48.8 to 54.7% (+11.9% relative change) over the 20-year period. However, after age standardization using the 2000–2004 population as standard, the increase was attenuated to 51.5% (+5.4%), indicating that approximately half of the observed crude trend was attributable to population aging (Figure 7A). Age standardization had minimal impact on co-recorded condition burden trends (Figure 7C). Endocrine/metabolic disease prevalence increased in both subtypes based on crude estimates: +19.8% in hemorrhagic and +9.9% in ischemic (age-standardized: +13.3% and +9.4%, respectively). The proportion of ischemic stroke patients with ≥3 co-recorded conditions rose from 24.8 to 33.3% (+34.2%). The heatmap (Figure 7D) visualizes these diverging trends. Full temporal trends, including age-standardized estimates, are presented in Supplementary Table S6.

**Figure 7:**
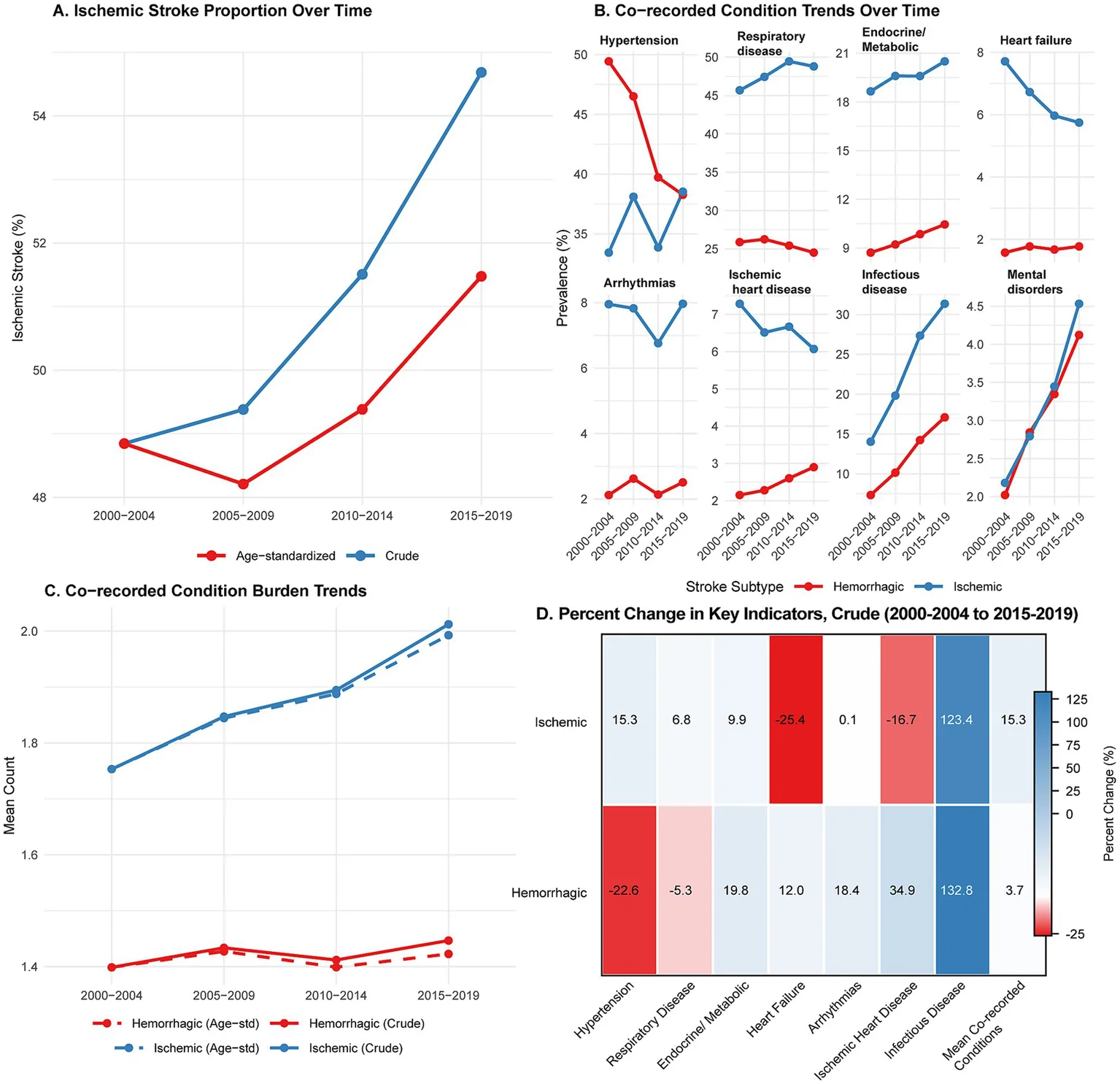
Temporal trends in stroke epidemiology (Brazil, 2000–2019). **(A)** Crude vs. age-standardized ischemic stroke proportion. **(B)** Co-recorded condition prevalence trends by subtype. **(C)** Crude vs. age-standardized mean co-recorded condition count. **(D)** Percent change heatmap of key indicators (crude percent change from 2000–04 to 2015–19; see Supplementary Table S6).

## 4 Discussion

This nationwide analysis of 598,186 natural deaths in Brazil over two decades revealed three main findings. First, hemorrhagic and ischemic stroke have fundamentally different co-occurrence patterns of co-recorded conditions: hemorrhagic stroke exhibited simpler co-occurrence patterns centered on hypertension together with infectious, respiratory, and endocrine/metabolic conditions, while ischemic stroke exhibited clustered cardiovascular, respiratory, and metabolic multimorbidity. Second, a co-occurrence pattern between mental disorders and hypertension was identified in hemorrhagic stroke (lift = 1.27, confidence = 53.9%). This pattern should be regarded as hypothesis-generating only: its support (1.75%) fell below the 2% support threshold used for ischemic stroke, and its lift dropped below 1 (0.98) in the subset restricted to deaths with at least one co-recorded condition. Its absence from the ischemic-stroke rule set therefore cannot by itself be interpreted as evidence of subtype specificity. Third, demographic characteristics together with co-recorded condition profiles distinguished stroke subtypes with moderate accuracy (AUC = 0.768), and temporal trends showed a progressive epidemiological transition toward ischemic stroke predominance (crude: 48.8–54.7%) over 20 years.

Our finding that ischemic stroke patients have higher co-recorded condition burden than hemorrhagic stroke patients aligns with prior research (3, 10). The INTERSTROKE study established hypertension as the dominant risk factor for both subtypes, with stronger associations for hemorrhagic stroke (7), consistent with our observation that hypertension, nervous system disease, and blood disease were the only co-recorded conditions more prevalent in hemorrhagic stroke (hypertension: 42.5% vs. 36.4%; nervous system disease: 16.9% vs. 7.9%; blood disease: 2.9% vs. 1.0%). Our data identified a mental disorders–hypertension co-occurrence pattern in hemorrhagic stroke, consistent with meta-analyses linking depression to stroke risk 16 and with studies linking psychological stress to stroke risk (35, 36). Recent cohort studies have reported associations between psychosocial stress and increased stroke risk (37), but none examined subtype-specific effects. Notably, the age-adjusted OR for mental disorders favored ischemic stroke (age-adjusted OR = 1.37, 95% CI 1.35–1.39) and was larger than the unadjusted OR (1.08, 95% CI 1.07–1.10); age adjustment therefore strengthened rather than attenuated the association between mental disorders and ischemic stroke, indicating that age differences did not explain this association. Consistent with this direction, mental disorders were documented more frequently in ischemic than in hemorrhagic stroke in every age stratum (Supplementary Table S2). The co-occurrence observed in hemorrhagic stroke may partly reflect differences in co-recorded condition documentation patterns, and future studies should investigate whether stress-induced sympathetic activation and cortisol dysregulation more directly affect cerebral vasculature integrity (38).

The distinct co-occurrence patterns of co-recorded conditions we identified—simple for hemorrhagic versus clustered for ischemic—extend previous multimorbidity research in stroke patients (10, 11) by using association rule mining to reveal structural relationships between conditions. Our machine learning model achieved performance comparable to stroke prediction models for fatal stroke developed in high-income settings (21, 22), which is notable given that we relied solely on death certificate data without clinical measurements. The temporal trends we observed—increasing ischemic stroke proportion and co-recorded condition burden—mirror epidemiological transitions documented in other middle-income countries undergoing rapid urbanization and lifestyle changes (4, 12), although the pace and drivers of these transitions vary across countries.

Our findings have direct relevance for public health policy and practice in Brazil. However, LMICs are heterogeneous in their disease burden, healthcare access, and diagnostic capacity; the extent to which these fatal-stroke findings apply to other LMICs warrants further investigation (39). First, the hypothesis-generating co-occurrence of mental disorders and hypertension identified in hemorrhagic stroke suggests that integrated mental health and hypertension management deserve further investigation; however, given the limited support for this rule, these findings should not yet be translated into subtype-specific clinical or policy recommendations. This pattern requires prospective validation before its public health significance can be assessed, given the substantial prevalence of depression and other common mental disorders in Brazil (40). Further research should investigate hypertension screening and management among patients with mental disorders, particularly in primary care settings where most mental health care is delivered in LMICs (41).

Second, the distinct co-occurrence patterns of co-recorded conditions suggest that future research should investigate subtype-specific strategies. For hemorrhagic stroke, further studies should evaluate the role of hypertension control and mental health management. For ischemic stroke, comprehensive cardiovascular risk factor management warrants further investigation. This aligns with the World Health Organization’s HEARTS technical package for cardiovascular disease management in primary care (42).

Third, our finding that co-recorded condition profiles, in combination with demographic characteristics, can distinguish stroke subtypes with moderate accuracy (AUC = 0.768) is hypothesis-generating only. This model was developed and validated using mortality data from deceased patients and has not been tested in living patients with acute stroke. As such, it should not be used for clinical decision-making, particularly in settings where CT is unavailable, given the catastrophic consequences of misclassifying hemorrhagic stroke as ischemic.

Fourth, the temporal trends we documented—increasing ischemic stroke predominance and co-recorded condition burden—highlight the need for health system adaptation. However, approximately half of the observed increase in ischemic stroke proportion was attributable to Brazil’s population aging, underscoring the importance of age-standardized epidemiological monitoring in rapidly aging populations, particularly in Brazil and other middle-income countries undergoing similar demographic transitions. Brazil’s epidemiological transition may necessitate a greater focus on ischemic stroke prevention and management, including diabetes control and multimorbidity care. The 34% increase in ischemic stroke patients with ≥3 co-recorded conditions over 20 years highlights the importance of further research into integrated care models (43).

### 4.1 Study design limitations

Several methodological limitations should be considered. First, this is a cross-sectional analysis of death certificate data, which cannot distinguish pre-existing conditions from acute stroke complications (e.g., respiratory infections may be post-stroke; dementia may reflect pre-existing cerebrovascular disease). This study therefore describes contemporaneous co-recorded diagnoses in fatal stroke cases, not etiological risk factors or prospective predictors.

Second, the findings are derived exclusively from fatal stroke cases and may not generalize to non-fatal strokes or survivors, who represent the majority of the stroke burden. Third, diagnostic practices and death certificate coding vary by region and over time. Improved CT/MRI availability, regional healthcare access disparities, and changes in ICD-10 coding practices may have influenced subtype classification and co-recorded condition documentation.

Fourth, mental disorder diagnoses on death certificates are underreported (2–6% vs. 20–30% in population-based estimates 17), which may introduce selection bias toward more severe or institutionalized cases. Reverse causation is also possible: mental disorders recorded at death could reflect post-stroke neuropsychiatric complications or the vascular depression hypothesis rather than pre-existing conditions. Fifth, unmeasured confounders—including psychotropic medication use (particularly SSRIs, which increase bleeding risk), healthcare access disparities, and socioeconomic status—could not be accounted for.

Sixth, exclusion of unspecified stroke (I64), the largest category in the original database, may introduce selection bias. I64 cases are more common in regions with limited diagnostic imaging and may differ systematically from confirmed subtypes. Regional analysis (Supplementary Figure S4) confirmed that hemorrhagic stroke proportion ranged from 56.1% (North) to 40.1% (South), consistent with geographic disparities in imaging availability and I64 coding rates. Seventh, mental disorders were analyzed as a single category (F00–F99) due to death certificate coding limitations. Substance use disorders and mood/anxiety disorders have distinct biological pathways—coagulopathy vs. HPA axis dysregulation—that cannot be distinguished in these data. Future studies should disaggregate these subcategories.

Eighth, the temporal trend analysis was based on only four 5-year periods, limiting statistical power for trend detection. Annual data were not available in the publicly released dataset. Ninth, to focus on interpretable positive co-occurrence, only rules in which all co-recorded conditions were present were retained in Table 1; Figure 3; rules involving the absence of a condition (e.g., {hypertension = No}) were excluded, because an absent diagnosis on a death certificate does not confirm the absence of disease and may reflect ascertainment or coding practices rather than biological co-occurrence.

The analyses were conducted by researchers based in China without Brazilian co-authors. Although we relied on published validation studies of SIM data quality and the prior work of Milan de Souza et al. to contextualize our findings, the absence of local collaborators may limit interpretation of Brazilian healthcare system nuances.

Future research should validate the mental disorders–hypertension association in hemorrhagic stroke using prospective cohort studies with clinical phenotyping and explore whether mental health management reduces hemorrhagic stroke risk (44).

## 5 Conclusion

Hemorrhagic and ischemic stroke have fundamentally different co-occurrence patterns of co-recorded conditions. Mental disorders represent a hypothesis-generating co-occurrence pattern in hemorrhagic stroke that warrants further investigation. These findings provide a basis for hypothesis testing in future prospective studies in Brazil. The applicability of these fatal-stroke findings to other LMICs requires further cross-country investigation, given the substantial heterogeneity in healthcare infrastructure and epidemiological profiles across LMIC settings.

## Data Availability

Publicly available datasets were analyzed in this study. This data can be found at: Zenodo repository; dataset: https://doi.org/10.5281/zenodo.16953930 (accession: 10.5281/zenodo.16953930) Primary data source: Brazilian Mortality Information System (SIM), https://datasus.saude.gov.br.
