# Supplementary material for: Co-recorded conditions in ischemic and hemorrhagic stroke: a 20-year national analysis of 598,186 natural deaths with machine learning
Source: Front Neurol. 2026 Sep 24;17:1854246. doi: 10.3389/fneur.2026.1854246 (PMC13645715; doi:10.3389/fneur.2026.1854246)
Supplement: Supplementary file 1 [file Supplementary_file_1.docx]

Supplementary Material

# 1 Supplementary Figures and Tables

## Supplementary Tables

**Supplementary Table S1. ICD-10 Codes for Co-recorded conditions Classification**

| **Co-recorded condition** | **ICD-10 Codes** |
| --- | --- |
| Hypertension | I10-I15 |
| Respiratory disease | J00-J99 |
| Genitourinary disease | N00-N99 |
| Endocrine/metabolic disease | E00-E90 |
| Nervous system disease | G00-G99 |
| Digestive disease | K00-K93 |
| Infectious disease | A00-B99 |
| Skin disease | L00-L99 |
| Peripheral vascular disease | I70-I79 |
| Arrhythmias | I47-I49 |
| Ischemic heart disease | I20-I25 |
| Blood disease | D50-D89 |
| Heart failure | I50 |
| Mental disorders | F00–F99 |

**Supplementary Table S2. Age-Stratified Demographic Profiles**

| **Characteristic** | **Young (<60 years)** | **Middle-aged (60-79 years)** | **Elderly (≥80 years)** |
| --- | --- | --- | --- |
| N | 167,053 | 265,118 | 166,015 |
| Hemorrhagic n (%) | 122,447 (73.3) | 116,796 (44.1) | 49,481 (29.8) |
| Ischemic n (%) | 44,606 (26.7) | 148,322 (55.9) | 116,534 (70.2) |
| Female % | 48.1 | 47.2 | 59.7 |
| Co-recorded condition count mean |  |  |  |
| Hemorrhagic | 1.24 | 1.49 | 1.5 |
| Ischemic | 1.79 | 1.99 | 1.9 |
| ≥3 Co-recorded conditions % |  |  |  |
| Hemorrhagic | 11.7 | 17.2 | 17.1 |
| Ischemic | 25.6 | 31.5 | 28.7 |
| Hypertension % |  |  |  |
| Hemorrhagic | 38.9 | 46.3 | 41.9 |
| Ischemic | 34.5 | 38.9 | 33.8 |
| Respiratory disease % |  |  |  |
| Hemorrhagic | 19.1 | 28.4 | 33.7 |
| Ischemic | 39 | 48.6 | 51.2 |
| Endocrine/metabolic % |  |  |  |
| Hemorrhagic | 6.8 | 12.2 | 11.1 |
| Ischemic | 18.1 | 22.6 | 16.9 |
| Heart failure % |  |  |  |
| Hemorrhagic | 0.8 | 2 | 3.2 |
| Ischemic | 3.9 | 6 | 7.6 |
| Arrhythmias % |  |  |  |
| Hemorrhagic | 1.1 | 2.8 | 4.3 |
| Ischemic | 4.6 | 7.4 | 9 |
| Mental disorders % |  |  |  |
| Hemorrhagic | 4.5 | 2.5 | 1.8 |
| Ischemic | 6.2 | 3.3 | 2.8 |

**Supplementary Table S3. Sex-Stratified Demographic Profiles**

| **Characteristic** | **Male** | **Female** |
| --- | --- | --- |
| N | 293,688 | 304,498 |
| Hemorrhagic n (%) | 142,071 (48.4) | 146,653 (48.2) |
| Ischemic n (%) | 151,617 (51.6) | 157,845 (51.8) |
| Age ≥60 years % | 70.5 | 73.6 |
| Hypertension % |  |  |
| Hemorrhagic | 41.9 | 42.9 |
| Ischemic | 34.9 | 37.7 |
| Respiratory disease % |  |  |
| Hemorrhagic | 27.2 | 23.6 |
| Ischemic | 50.3 | 46.2 |
| Endocrine/metabolic % |  |  |
| Hemorrhagic | 9 | 10.4 |
| Ischemic | 17.7 | 21.8 |
| Heart failure % |  |  |
| Hemorrhagic | 1.8 | 1.7 |
| Ischemic | 5.9 | 6.7 |
| Arrhythmias % |  |  |
| Hemorrhagic | 2.3 | 2.4 |
| Ischemic | 6.7 | 8.5 |
| Mental disorders % |  |  |
| Hemorrhagic | 4.3 | 2.2 |
| Ischemic | 4.5 | 2.6 |
| Infectious disease % |  |  |
| Hemorrhagic | 14.2 | 12 |
| Ischemic | 25.9 | 24.5 |

**Supplementary Table S4. Stratified Model Performance by Age and Sex**

| **Stratum** | **Group** | **N** | **AUC** | **Accuracy** | **Sensitivity** | **Specificity** |
| --- | --- | --- | --- | --- | --- | --- |
| Age | Age <60 years | 167,053 | 0.717 (0.711-0.722) | 0.756 | 0.949 | 0.226 |
| Age | Age 60-79 years | 265,118 | 0.710 (0.707-0.714) | 0.668 | 0.563 | 0.75 |
| Age | Age ≥80 years | 166,015 | 0.684 (0.680-0.690) | 0.717 | 0.183 | 0.943 |
| Sex | Male | 293,688 | 0.727 (0.724-0.730) | 0.673 | 0.656 | 0.69 |
| Sex | Female | 304,498 | 0.748 (0.745-0.751) | 0.69 | 0.677 | 0.703 |

Note: Hemorrhagic stroke was the positive class; metrics were evaluated at the 0.5 decision threshold. AUC, area under the ROC curve.

**Supplementary Table S5. Key Association Rule Robustness (Mental Disorders → Hypertension)**

| **Dataset** | **Support(%)** | **Confidence(%)** | **Lift** |
| --- | --- | --- | --- |
| Natural Deaths Only (main analysis) | 1.75 | 53.9 | 1.27 |
| ≥1 Co-recorded conditions | 2.26 | 53.9 | 0.98 |
| ≥2 Co-recorded conditions | 4.15 | 63.9 | 1.05 |
| Excluding respiratory disease | 1.88 | 56.9 | 1.29 |
| Excluding infectious disease | 1.86 | 56.1 | 1.26 |

**Supplementary Table S6. Full Temporal Trends Data (2000-2019) with Age-Standardized Estimates**

| **Period** | **Subtype** | **N** | **Hypertension** | | **Respiratory** | | Endocrine/ metabolic | | **Heart failure** | | **Arrhythmias** | | **Ischemic heart disease** | | **Infectious** | | ≥3 CC | | Mean CC | |
| --- | --- | --- | --- | --- | --- | --- | --- | --- | --- | --- | --- | --- | --- | --- | --- | --- | --- | --- | --- | --- |
|  |  |  | **Crude** | **AS** | **Crude** | **AS** | **Crude** | **AS** | **Crude** | **AS** | **Crude** | **AS** | **Crude** | **AS** | **Crude** | **AS** | **Crude** | **AS** | **Crude** | **AS** |
| 2000-2004 | Hemorrhagic | 51,950 | 49.44 | 49.95 | 25.88 | 27.92 | 8.72 | 9.45 | 1.58 | 1.96 | 2.12 | 2.50 | 2.15 | 2.47 | 7.34 | 7.84 | 13.12 | 13.93 | 1.36 | 1.40 |
| 2000-2004 | Ischemic | 49,606 | 33.43 | 34.08 | 45.68 | 43.92 | 18.65 | 18.48 | 7.71 | 6.93 | 7.95 | 7.35 | 7.29 | 7.02 | 14.03 | 14.33 | 24.82 | 24.40 | 1.77 | 1.75 |
| 2005-2009 | Hemorrhagic | 62,092 | 46.51 | 47.24 | 26.26 | 27.77 | 9.22 | 9.77 | 1.77 | 2.03 | 2.62 | 2.93 | 2.28 | 2.52 | 10.15 | 10.70 | 14.38 | 15.02 | 1.40 | 1.43 |
| 2005-2009 | Ischemic | 60,574 | 38.11 | 37.88 | 47.43 | 45.55 | 19.60 | 19.49 | 6.73 | 6.15 | 7.83 | 7.15 | 6.52 | 6.25 | 19.82 | 19.69 | 27.78 | 27.11 | 1.87 | 1.84 |
| 2010-2014 | Hemorrhagic | 79,601 | 39.73 | 40.28 | 25.43 | 26.68 | 9.86 | 10.33 | 1.67 | 1.86 | 2.14 | 2.35 | 2.60 | 2.77 | 14.24 | 14.82 | 14.52 | 15.02 | 1.37 | 1.40 |
| 2010-2014 | Ischemic | 84,550 | 33.87 | 33.21 | 49.45 | 47.16 | 19.60 | 19.42 | 5.97 | 5.33 | 6.76 | 6.05 | 6.67 | 6.57 | 27.34 | 27.01 | 28.58 | 27.88 | 1.92 | 1.89 |
| 2015-2019 | Hemorrhagic | 95,081 | 38.27 | 38.67 | 24.52 | 25.17 | 10.45 | 10.71 | 1.77 | 1.86 | 2.51 | 2.65 | 2.90 | 2.98 | 17.09 | 17.38 | 16.45 | 16.73 | 1.41 | 1.42 |
| 2015-2019 | Ischemic | 114,732 | 38.53 | 37.21 | 48.79 | 45.97 | 20.50 | 20.22 | 5.75 | 5.15 | 7.96 | 6.96 | 6.07 | 6.06 | 31.34 | 30.75 | 33.31 | 32.08 | 2.04 | 1.99 |

Abbreviations: Hyp., hypertension; Resp., respiratory disease; Endo/Met., endocrine/metabolic disease; HF, heart failure; Arr., arrhythmias; IHD, ischemic heart disease; Infect., infectious disease; CC, co-recorded conditions; AS, age-standardized.

Note: Age-standardized estimates (AS) were calculated by direct standardization using the 2000-2004 age distribution of all natural-death stroke cases as the standard population (age groups: 19-39, 40-59, 60-79, ≥80 years). Crude ischemic proportion increased from 48.8% (2000-2004) to 54.7% (2015-2019); after age standardization, the increase was attenuated to 51.5%, indicating that approximately half of the crude temporal increase was attributable to population aging. Values are reported to two decimal places, allowing direct reproduction of the percent changes shown in Figure 7D (e.g., hypertension in hemorrhagic stroke: (38.27 − 49.44)/49.44 = −22.6%).

## Supplementary Figures

**Supplementary Figure S1. Sensitivity Analysis Results**

(A) Forest plot of AUC with 95% CIs across five sensitivity analyses. (B) Performance metrics (AUC, accuracy, sensitivity, specificity) by analysis.

**Supplementary Figure S2. Age-Stratified Analysis:**

(A) Stroke subtype distribution by age. (B) Co-recorded condition prevalence by age and subtype. (C) Model AUC by age group. (D) Top 5 predictors by age group.

**Supplementary Figure S3. Sex-Stratified Analysis**

(A) Stroke subtype distribution by sex. (B) Co-recorded condition prevalence by sex and subtype. (C) Model AUC by sex. (D) Top 5 predictors by sex. EMD, endocrine/metabolic disease; IHD, ischemic heart disease; MD, mental disorders.

Supplementary Figure S4. Regional Sensitivity Analysis

(A) Hemorrhagic stroke proportion by Brazilian region. (B) Mean Co-recorded condition count by region and stroke subtype.
